# Dll4 Blockade Potentiates the Anti-Tumor Effects of VEGF Inhibition in Renal Cell Carcinoma Patient-Derived Xenografts

**DOI:** 10.1371/journal.pone.0112371

**Published:** 2014-11-13

**Authors:** Kiersten Marie Miles, Mukund Seshadri, Eric Ciamporcero, Remi Adelaiye, Bryan Gillard, Paula Sotomayor, Kristopher Attwood, Li Shen, Dylan Conroy, Frank Kuhnert, Alshad S. Lalani, Gavin Thurston, Roberto Pili

**Affiliations:** 1 Genitourinary Program, Roswell Park Cancer Institute, Buffalo, New York, United States of America; 2 Department of Pharmacology & Therapeutics, Roswell Park Cancer Institute Division, University at Buffalo, Buffalo, New York, United States of America; 3 Department of Cancer Pathology & Prevention, Roswell Park Cancer Institute Division, University at Buffalo, Buffalo, New York, United States of America; 4 Department of Molecular and Cellular Biology, Roswell Park Cancer Institute Division, University at Buffalo, Buffalo, New York, United States of America; 5 Department of Biostatistics & Bioinformatics, Roswell Park Cancer Institute Division, University at Buffalo, Buffalo, New York, United States of America; 6 Medicine and Experimental Oncology, University of Turin, Turin, Italy; 7 Regeneron Pharmaceuticals, Inc., Tarrytown, New York, United States of America; University of Kentucky College of Medicine, United States of America

## Abstract

**Background:**

The Notch ligand Delta-like 4 (Dll4) is highly expressed in vascular endothelium and has been shown to play a pivotal role in regulating tumor angiogenesis. Blockade of the Dll4-Notch pathway in preclinical cancer models has been associated with non-productive angiogenesis and reduced tumor growth. Given the cross-talk between the vascular endothelial growth factor (VEGF) and Delta-Notch pathways in tumor angiogenesis, we examined the activity of a function-blocking Dll4 antibody, REGN1035, alone and in combination with anti-VEGF therapy in renal cell carcinoma (RCC).

**Methods and Results:**

Severe combined immunodeficiency (SCID) mice bearing patient-derived clear cell RCC xenografts were treated with REGN1035 and in combination with the multi-targeted tyrosine kinase inhibitor sunitinib or the VEGF blocker ziv-aflibercept. Immunohistochemical and immunofluorescent analyses were carried out, as well as magnetic resonance imaging (MRI) examinations pre and 24 hours and 2 weeks post treatment. Single agent treatment with REGN1035 resulted in significant tumor growth inhibition (36–62%) that was equivalent to or exceeded the single agent anti-tumor activity of the VEGF pathway inhibitors sunitinib (38–54%) and ziv-aflibercept (46%). Importantly, combination treatments with REGN1035 plus VEGF inhibitors resulted in enhanced anti-tumor effects (72–80% growth inhibition), including some tumor regression. Magnetic resonance imaging showed a marked decrease in tumor perfusion in all treatment groups. Interestingly, anti-tumor efficacy of the combination of REGN1035 and ziv-aflibercept was also observed in a sunitinib resistant ccRCC model.

**Conclusions:**

Overall, these findings demonstrate the potent anti-tumor activity of Dll4 blockade in RCC patient-derived tumors and a combination benefit for the simultaneous targeting of the Dll4 and VEGF signaling pathways, highlighting the therapeutic potential of this treatment modality in RCC.

## Introduction

Kidney cancer strikes close to 65,000 Americans every year and kills over 13,000 [1]. Renal cell carcinoma (RCC) is the most common type of kidney cancer, with 80% diagnosed as clear cell (cc) RCC. Treatment of localized RCC is usually centered on surgery and immunotherapy. Unfortunately, approximately 30–40% of kidney cancer patients eventually develop metastatic RCC and the current treatment options are limited. The well-vascularized nature of RCC has generated considerable interest in the development of anti-angiogenic therapies for this disease.

Vascular endothelial growth factor (VEGF) is a protein that stimulates vasculogenesis and angiogenesis by initiating blood vessel sprouting and endothelial proliferation. Overexpression of VEGF is often associated with tumor growth and metastases and is a common target for cancer therapy [2]. Several anti-VEGF therapies, including tyrosine kinase inhibitors (TKIs), are currently used in the frontline management of RCC. Sunitinib is an oral, multi-targeted receptor TKI that is FDA approved for the treatment of RCC and GIST; and which has been shown to inhibit tumor vascularization by diminishing signaling through VEGF receptors 1 and 2, and platelet derived growth factor receptor (PDGFR). Ziv-aflibercept is a protein therapeutic that binds to all isoforms of VEGF-A, as well as VEGF-B and placental growth factor (PlGF) [3], [4]. In several types of tumor xenograft models, including RCC, ziv-aflibercept was found to inhibit tumor growth with an associated large reduction of tumor vasculature, with less promotion of changes in gene expression in normal organs than seen following receptor TKI treatment [5], [6]. Ziv-aflibercept was recently approved for use in combination with chemotherapy for the treatment of colon carcinoma in patients who previously failed oxaliplatin-based therapy [7]. Further, ziv-aflibercept is currently under exploratory clinical investigations in patients with clear cell RCC who are refractory to VEGF-tyrosine kinase inhibitors (NCI trial number E4805). Unfortunately, the clinical benefit associated with anti-VEGF therapies is often limited, as patients exhibit acquired tumor resistance to VEGF inhibition; thus there is great interest in identifying additional angiogenesis targets that, in combination with anti-VEGF therapies, can lead to more effective treatments for RCC.

The Dll4-Notch pathway is an evolutionarily conserved signaling pathway that functions as a key negative regulator of physiological and pathological angiogenesis downstream of VEGF [8]. Dll4 is a Notch ligand that is induced in endothelial tip cells of angiogenic sprouts and loss of expression has been shown to lead to excessive production of aberrant non-functional tumor vessels and associated reduced tumor growth [9], [10]. Dll4 is predominately found in the developing endothelium, with an almost 9-fold increased expression reported within the vasculature of ccRCC, as compared to normal kidneys [11]. Multiple tumor types have been found to express Dll4 and RCC, in particular, has been shown to be regulated by the Dll4/Notch pathway [12]. Accordingly, therapeutic targeting of Dll4 in the treatment of renal cell carcinoma may hold much promise.

The aim of this study was to explore the effects of Dll4 antibody therapy alone and in combination with approved VEGF inhibitors on tumor growth and perfusion in patient-derived xenograft (PDX) ccRCC models. In our experimental models, we observed considerable single agent activity of Dll4 antibody targeting stromal Dll4, and an enhancement of the anti-tumor effects by combination with VEGF inhibition. In both cases, the anti-tumor effects were associated with profound tumor vascular changes and reduction in tumor perfusion. In addition, the anti-tumor efficacy of anti-Dll4 and anti-VEGF combination therapy was also observed in a sunitinib resistant ccRCC model, which further highlights the great therapeutic potential of targeting these two pathways.

## Materials and Methods

### Compounds

REGN1035, REGN421 (also known as enoticumab), and ziv-aflibercept (also known as VEGF Trap) were produced by Regeneron Pharmaceuticals, Inc. (Tarrytown, NY). REGN1035 is a preclinical monoclonal mouse antibody that selectively binds and blocks murine Dll4. REGN421 is a fully human IgG1 monoclonal antibody that binds human Dll4 and is currently under clinical investigation in a Phase I study in patients with advanced solid tumor malignancies. Ziv-aflibercept is a fully human fusion protein comprised of the extracellular domains of VEGFR1 and VEGFR2 fused to a human IgG1 constant domain. Sunitinib (Sutent) was purchased from LC Laboratories (Woburn, MA).

### Xenograft models and treatment protocol

RP-R-01 is a xenograft model established from a skin metastasis from a patient with sporadic ccRCC, previously treated with sunitinib. VHL (von Hippel-Lindau) was not found to be expressed in RP-R-01 tumor cells. The establishment and characterization of this model was previously described [13]. RP-R-02 is a xenograft model also established from a skin metastasis, but from a patient with VHL syndrome and hereditary ccRCC, with no prior treatments. Importantly, both models were passaged only *in vivo*, thus minimizing the selection for growth and loss of cellular heterogeneity of the primary tumor often seen in cell culture. Collection of tumor samples was obtained via regulatory approval at the institution. The *in vivo* experiments were conducted once.

Immunodeficient SCID male mice purchased from Roswell Park Cancer Institute (RPCI) were utilized for these studies and all procedures were approved by The Institute Animal Care and Use Committee (IACUC). Mice were kept in a temperature controlled room on a 12/12 hours light/dark schedule with food and water *ad libitum*. Mice were implanted subcutaneously in the flank area with ∼0.5 mm^3^ size RP-R-01 or RP-R-02 ccRCC tumor tissue. Approximately 6 weeks later, when tumors reached an average volume of 68.0 mm^3^, mice were divided into homogenous groups (7–8 mice/group) as determined by caliper measurements. Tumor volume was calculated as mm^3^ (√(length × width))^3^ ×0.5. Mice were treated with vehicle (hFc control protein, 4.89 mg/kg, s.q.), sunitinib (40 mg/kg, 5x/wk, orally), or ziv-aflibercept (5 mg/kg, 2x/wk, s.q.) and/or REGN1035 (5 mg/kg, 1x/wk, s.q.) for a period of five to six weeks.

A sunitinib resistant ccRCC model was established utilizing subcutaneous implantation of RP-R-01 tumor tissue. Mice with established tumors (average volume of 78.0 mm^3^) were treated with sunitinib (40 mg/kg, 5x/wk, orally) for 4 weeks until tumor tissue was no longer responsive to treatment (tumor volume was ∼6 times that of pretreatment volume). At which time, mice were treated with either sunitinib (40 mg/kg, 5x/wk, orally), ziv-aflibercept (5 mg/kg, 2x/wk, s.q.), REGN1035 (5 mg/kg, 1x/wk, s.q.) or the combination of ziv-aflibercept plus REGN1035 for a period of four weeks.

Mouse body weight and tumor caliper measurements were taken weekly. Body weight changes over the course of treatments are given in Table S1. Some weight loss was observed in mice treated with REGN1035 alone and in combination with sunitinib, ziv-aflibercept or REGN421. The vehicle group in 1 of 4 experiments also showed a reduction in weight, most likely due to tumor growth. At the time of harvest, it was noted that the livers of some (∼40%) of the mice treated with REGN1035 alone and in combination with ziv-aflibercept appeared patchy. Upon histological examination, the livers showed evidence of heterogeneous septal fibrosis and vascular congestion (Fig. S1). These findings are in agreement with a previous report on the effects of Dll4 blockade on rodent liver histology [14]. Tumor tissue was harvested and weighed at the end of treatment, and fixed in 10% normal buffered formalin or zinc.

### Immunohistochemistry

Formalin fixed and processed tissue sections were embedded in paraffin blocks and cut at 4 µm, placed on charged slides, and dried at 60°C for one hour. Slides were cooled to room temperature, deparaffinized in three changes of xylene, and rehydrated using graded alcohols. For antigen retrieval, slides were treated by citrate buffer (pH 6.0) (Biocare Medical) and heated in the microwave for 10 minutes, followed by a 15 minute cool down. Endogenous peroxidase was quenched with aqueous 3% H_2_O_2_ for 10 minutes and washed with PBS/T. Slides were loaded on a DAKO autostainer and serum free protein block (DAKO) was applied for 5 minutes, blown off, and the antibodies (Ki67, Thermo Scientific; CD31, Dianova) were applied for one hour. Biotinylated goat anti-rabbit IgG (Vector) secondary antibody was applied for 30 minutes, followed by the Elite ABC Kit (Vectastain), also for 30 minutes and the DAB chromogen (Dako) for five minutes, OR biotinylated goat anti-rat IgG (BD Pharmingen) secondary-thirty minutes, followed by ZSA-(Streptavidin Horseradish Peroxidase Conjugate (Invitrogen) for thirty minutes followed by the DAB chromogen for five minutes. Finally, the slides were counterstained with hematoxylin, dehydrated, cleared and cover slipped. Images were captured using a Scanscope XT system (Aperio Imaging) and analyzed using Imagescope software (Aperio). Necrosis (H&E), Ki67, and CD31 immunostaining quantification, approximately 4 randomly selected fields of 6 to 8 samples per treatment, was done in a blind manner using Image J software. Results are expressed as the average per treatment of positive area (H&E and CD31) and positive nuclei (Ki67) ± S.E.

### Immunofluorescence

Tumor tissue was removed and fixed in formalin for 24 hours. Samples were dehydrated and paraffin embedded. Paraffin blocks were sectioned (6 µm), deparaffinized, and antigen retrieval was performed by boiling the slides for 15 minutes in citrate buffer (pH 6.0). Slides were blocked in 1% bovine serum albumin (Sigma-Aldrich), diluted in PBS for 30 minutes, and incubated overnight with the primary anti-mouse CD31 antibody (1/40, Dianova), followed by incubation with the secondary antibody Cy3-conjugated anti-rat IgG (1/400, Invitrogen). Sections were mounted using Vectashield (Vector Laboratories). CD31 immunostaining quantification, approximately 4 randomly selected fields of 6 to 8 samples per treatment, was done in a blind manner using Image J software. Background was subtracted to determine the percentage of positive area. Results are expressed as the average per treatment of CD31 positive area ± S.E.

### Magnetic Resonance Imaging (MRI)

Experimental MRI examinations were carried out in a 4.7 T/33-cm horizontal bore magnet (GE NMR Instruments, Fremont, CA) incorporating AVANCE digital electronics (Bruker Biospec with ParaVision 3.0.2; Bruker Medical Inc., Billerica, MA) and a removable gradient coil insert (G060, Bruker Medical Inc., Billerica, MA) generating maximum field strength of 950 mT/m and a custom-designed 35-mm RF transmit-receive coil. Mice were placed on a form-fitted MR-compatible sled (Dazai Research Instruments, Toronto, Canada) and supplied with 2% isoflurane during image acquisition. Respiration rates and core-body temperature were monitored continuously while mice were in the scanner. Preliminary scout images were acquired on the sagittal and axial planes to assist in slice prescription for subsequent scans. Multislice non contrast-enhanced T2-weighted images were acquired on the axial planes with the following parameters: TE_eff_ = 41 ms, TR = 2500 ms, FOV  = 3.2×3.2 cm, matrix size  = 256×192, 25 slices, slice thickness 1 mm). T1-relaxation rates (R1) were measured using a saturation recovery, fast spin echo as described [15], [16]. A series of ten images (3 before contrast and 7 following administration of albumin-GdDTPA, 0.1 mmol/kg) were obtained to estimate changes in relative blood volume and permeability of tumors. MR angiography was performed using a three-dimensional spoiled gradient echo sequence (matrix size 192×96×96; FOV 4.8×3.2×3.2 cm, flip angle  = 40°, acquisition time  = 2 m 18 s. Raw image sets were transferred to a processing workstation and converted into Analyze format (Analyze 7.0, Analyze Direct, Overland Park, KS, USA). Linear regression analysis of the normalized change in R1 versus time curve was carried out to compute the relative blood volume of tumors. T1 relaxation maps (R1 maps) of animals were calculated on a pixel-by-pixel basis in MATLAB (Math Works, Inc.). For each treatment group, T1 enhancement maps were generated by subtracting a postcontrast R1 map from the pre contrast R1 map of the same animal.

### Statistical Analysis

Data are expressed as mean ± standard error (S.E). Tumor size is modeled as a function of time, experimental group and their interaction using linear mixed models, with tumor growth rates compared between experimental groups using the appropriate contrasts of estimated interaction terms. The association between end of treatment (EOT) outcomes and experimental group are evaluated using ANOVA models, with pairwise comparisons made using two-sample t-tests when appropriate. All tests are two-sided. All model assumptions were verified graphically and a log-transformation was found to be necessary for tumor size. All pairwise comparisons were adjusted using the Holm-Bonferroni method for controlling experiment wise error-rate. The analyses were conducted in Graph Pad Prism 5 (Graph Pad Software Inc.) and SAS v9.4 (Cary, NC) at a statistical significance level of 0.05.

## Results

### Potent anti-tumor activity of Dll4 blockade in RCC PDX models plus a significant combination benefit for combined inhibition of Dll4 and VEGF signaling

**Figure 1:**
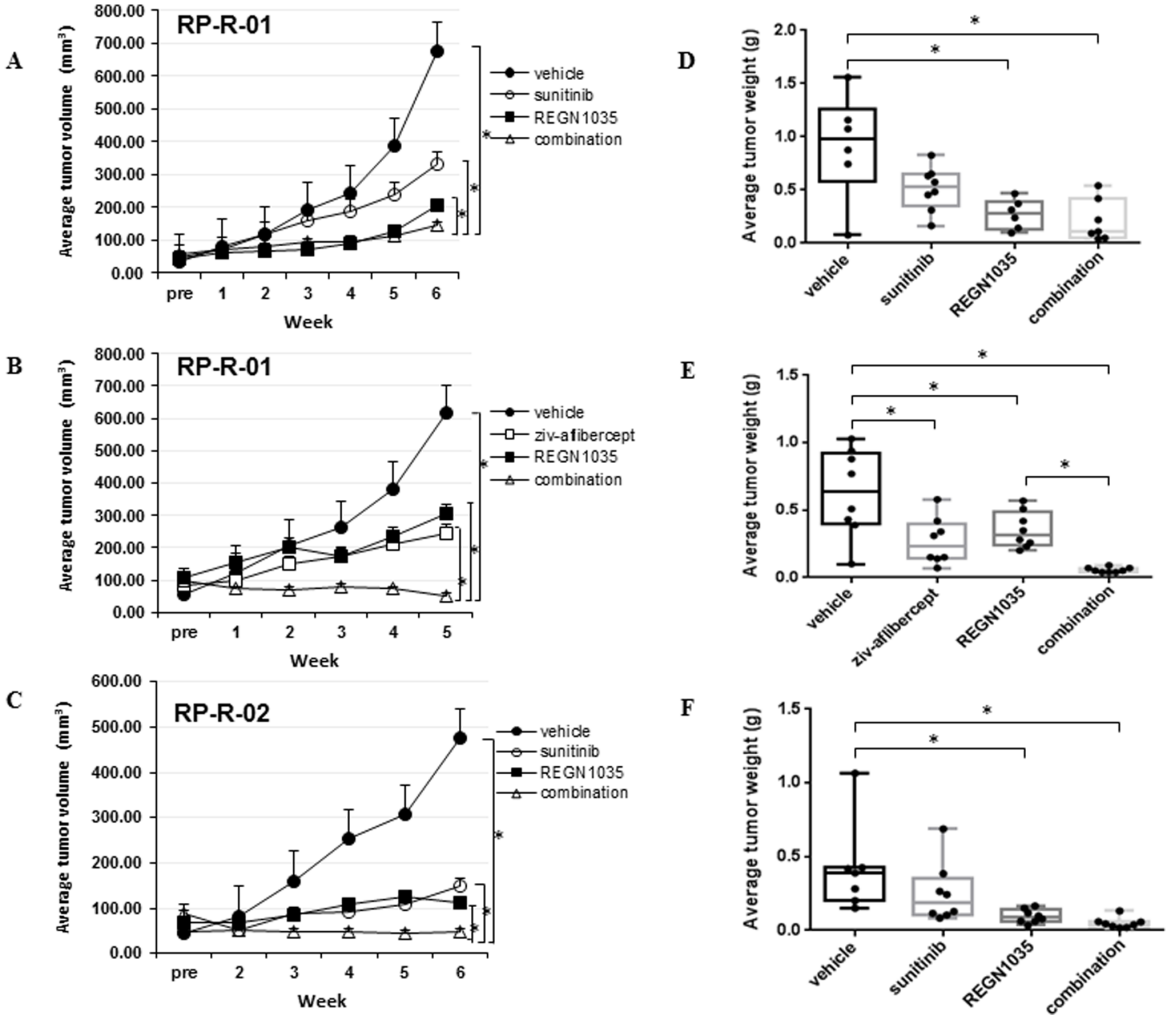
Anti-tumor efficacy of anti-Dll4 (REGN1035) and/or anti-VEGF (sunitinib or ziv-aflibercept) in RP-R-01 and RP-R-02 ccRCC patient derived xenografts. Mice (8 mice/group) were treated with vehicle, sunitinib, or ziv-aflibercept and/or REGN1035. Left panel: Tumor growth curves. (A) RP-R-01 REGN1035 and/or sunitinib treatment groups (B) RP-R-01 REGN1035 and/or ziv-aflibercept treatment groups (C) RP-R-02 REGN1035 and/or sunitinib treatment groups. Each line represents the average tumor volume (mm^3^) of each treatment group ± S.E. (D, E, F) End point tumor weights (g). * *p*<0.05 using adjusted t-test analysis.

To assess the anti-tumor efficacy of anti-Dll4 and anti-Dll4/VEGF combination therapy, SCID mice were implanted with RP-R-01 or RP-R-02 ccRCC patient-derived xenograft tumors and treated with REGN1035 and/or sunitinib or ziv-aflibercept (Fig. 1A–C). Single agent treatment in the RP-R-01 tumor model reduced tumor growth by 38% (sunitinib); 46% (ziv-aflibercept); 36% and 55% (REGN1035), *p*<0.01 vs. vehicle, and an even more significant effect on tumor growth was observed in combination treated groups (72% reduction with REGN1035 plus sunitinib; 80% reduction with REGN1035 plus ziv-aflibercept, *p*<0.01 combination vs. vehicle, *p*<0.01 combination vs. single agent; Table S1). Noteworthy, REGN1035 treatment combined with ziv-aflibercept resulted in not only an additive anti-tumor effect, but also regression of established RP-R-01 tumors (Fig. 1B). A similar observation was found in the treatment-naïve patient-derived RP-R-02 model, with 38% (sunitinib) and 55% (REGN1035) reduction of growth from single agent treatment and 72% reduction following combination treatment, *p*<0.01 (Fig. 1C). At end of treatment (EOT), the average RP-R-01 tumor volume and weight following REGN1035 and/or sunitinib administration are shown in Figures 1A and 1D, respectively: vehicle (677.3 mm^3^, 0.914 g), sunitinib (329.5 mm^3^, 0.512 g), REGN1035 (204.3 mm^3^, 0.271 g), and combination (144.6 mm^3^, 0.210 g). Figures 1B and 1E show the average EOT tumor volume and weight, respectively, of RP-R-01 tissue treated with REGN1035 and/or ziv-aflibercept: vehicle (617.7 mm^3^, 0.631 g), ziv-aflibercept (245.4 mm^3^, 0.270 g), REGN1035 (307.9 mm^3^, 0.353 g), and combination (52.5 mm^3^, 0.056 g). RP-R-02 tumor volume and weight are shown in Figures 1C and 1F, respectively: vehicle (476.3 mm^3^, 0.419 g), sunitinib (149.3 mm^3^, 0.251 g), REGN1035 (110.31 mm^3^, 0.096 g), and combination (47.5 mm^3^, 0.049 g). ANOVA analysis showed a significant association between treatment and EOT tumor weight, with all *p* values <0.05. The tumor growth rate was lower in the combination treatment than in single agent or vehicle. The single agents have no significant difference, but both have lower growth rates than the vehicle. Overall, all treatments were well tolerated. No overt signs of toxicity (i.e. extreme weight loss, lack of food consumption or diarrhea) were observed in either xenograft model following REGN1035 and/or sunitinib treatment. A 16% decrease in mouse body weight relative to vehicle was observed following REGN1035 plus ziv-aflibercept combination treatment in the RP-R-01 model. However, in that experiment the vehicle group also had a 10% decrease in body weight and the difference with the combination group was not statistically significant (Table S2). The mice otherwise appeared healthy with no other signs of distress.

**Figure 2:**
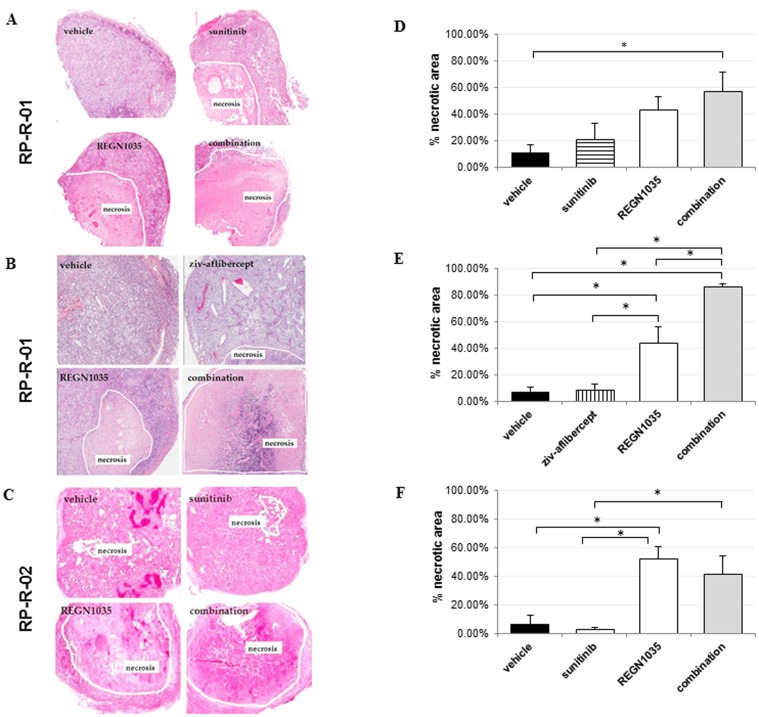
Effect of REGN1035 and/or anti-VEGF (sunitinib or ziv-aflibercept) treatment on tumor cell viability. Mice (8 mice/group) implanted with (A, B) RP-R-01 or (C) RP-R-02 tissues were treated with vehicle, sunitinib, or ziv-aflibercept and/or REGN1035. Tumors were harvested, processed, and tissue sections were stained for hematoxylin and eosin. Left panels: Representative images of tumor necrosis. (D, E, F) Quantitative analysis was done in a blinded fashion. Results are expressed as mean percentage necrotic area ± S.E. *p<0.05 using adjusted t-test analysis.

To examine the effects of treatment on tumor necrosis, end of treatment tumor tissue sections were stained with hematoxylin and eosin (H&E). As shown in Figures 2A–C, a modest reduction in viable tissue was observed following single agent treatment with anti-VEGF therapy (sunitinib, 3–21% necrosis), (ziv-aflibercept, 9% necrosis). Tumors treated with REGN1035 or combinations displayed a substantially increased percentage of necrosis than vehicle or VEGF inhibitor-treated tumors (vehicle, 7–11%; REGN1035, 44–52%; REGN1035 plus sunitinib, 41–57%; REGN1035 plus ziv-aflibercept, 86%, all *p*<0.05 vs. vehicle) (Fig. 2D–F).

**Figure 3:**
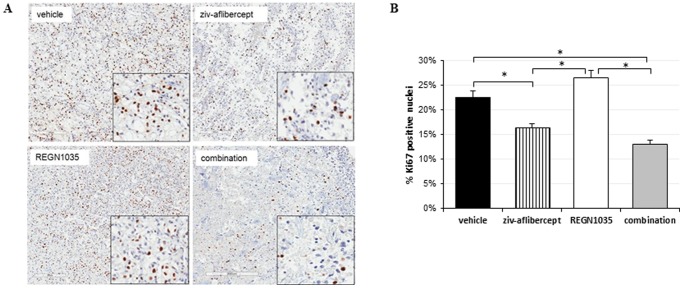
Effects of REGN1035 and/or ziv-aflibercept on tumor cell proliferation in RP-R-01 ccRCC xenograft. Mice were treated with vehicle, ziv-aflibercept and/or REGN1035. Tumors were harvested, processed, and tissue sections were stained for differential expression of Ki67. (A) Representative images. (B) Quantitative analysis was done in a blinded manner. Results are expressed as mean percentage positive nuclei ± S.E. *p<0.05 using adjusted t-test analysis.

We were also interested in assessing the effect of anti-Dll4 and anti-VEGF treatment on cellular proliferation. As shown in Figures 3A–B, single agent inhibition of Dll4 signaling with REGN1035 induced a modest increase of Ki67 expression in RP-R-01 tumor sections, presumably reflecting increased endothelial proliferation. In contrast, ziv-aflibercept and the combination with REGN1035 was associated with a significant decrease in cellular proliferation (ziv-aflibercept, 28% reduction, *p*<0.05; combination, 42% reduction, *p*<0.01, as compared to vehicle), which correlates with the inhibition of tumor growth shown in Figure 1B.

### Dll4 blockade enhances the anti-vascular effects of VEGF inhibition

**Figure 4:**
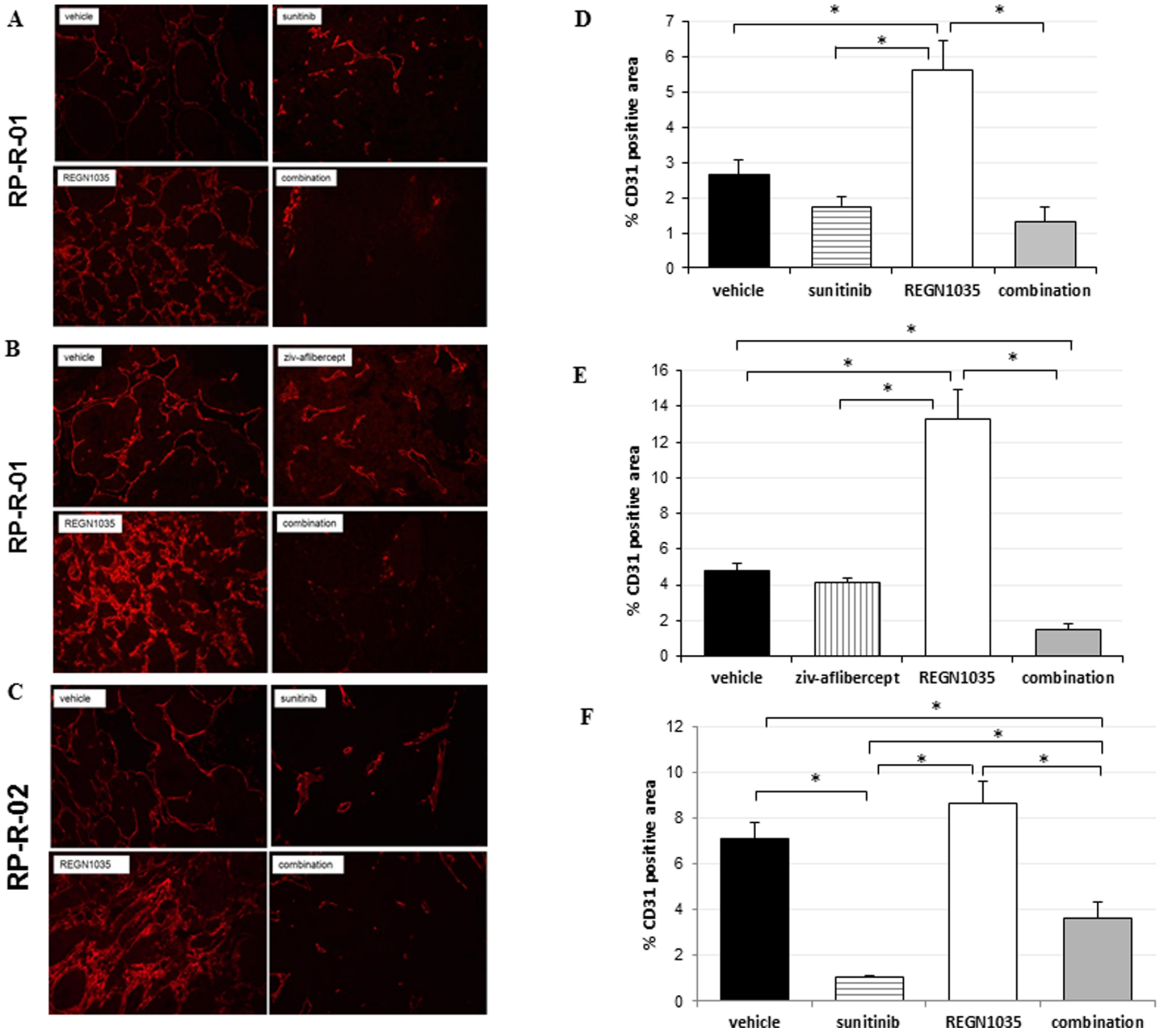
Effect of REGN1035 and/or anti-VEGF (sunitinib or ziv-aflibercept) on (A, B) RP-R-01 and (C) RP-R-02 tumor vasculature. Tumors from treated mice were harvested, processed, and tissue sections were stained for the differential expression of CD31 (red) for visualization of endothelial cells (left panels). (D, E, F) Blinded quantitative analysis of CD31 (right panels). Results are expressed as mean percentage positive stained area ± S.E. *p<0.05 using adjusted t-test analysis.

We assessed the anti-vascular effects of anti-Dll4 and anti-VEGF treatment in our RCC models using immunofluorescence, immunohistochemistry, and non-invasive magnetic resonance imaging (MRI). The panel of images shown in Figure 4 represents immunofluorescence staining of end-of-treatment tumor sections [RP-R-01 (A and B), RP-R-02 (C)] with anti-CD31 antibody. A reduction in vessel density was observed following single agent treatment with sunitinib (34% and 85% reduction in RP-R-01, *p*<0.01 and RP-R-02, respectively) and ziv-aflibercept (14% reduction in RP-R-01) (Fig. 4D–F). Tumors treated with single agent REGN1035, in contrast, showed a statistically significant increase in vascular structures, as compared to vehicle (111% and 180% increase in RP-R-01, *p*<0.01; 22% increase in RP-R-02). This increase in tumor microvascular density is consistent with the observed increase in Ki67 staining shown in Figure 3. In addition, REGN1035 treatment promoted structural changes within the tumor vasculature with the appearance of extensively unorganized aberrant networks of small, highly branched vessels. Combination treatment resulted in significantly less tumor vascularity (50% and 68% reduction in RP-R-01; 49% reduction in RP-R-02, *p*<0.05), with a great percentage of the tissue nearly devoid of vascular networks.

**Figure 5:**
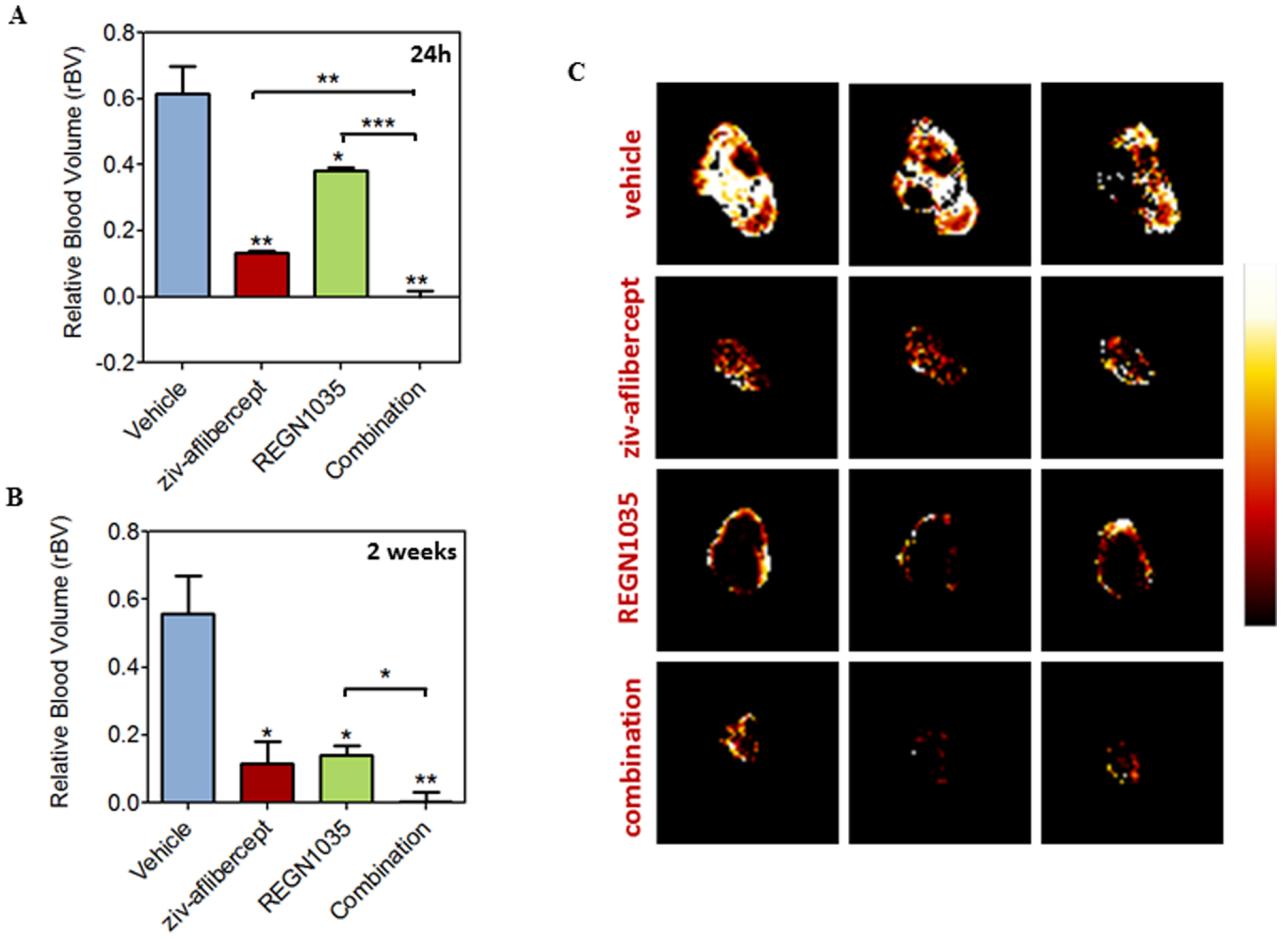
Vascular response of RP-R-01 tumors to combined DLL4-VEGF blockade. MRI-based estimates of relative blood volume estimates of RP-R-01 tumors at (A) 24 hours and (B) 2 weeks (C) Panel of images represent contrast enhancement maps of a representative tumor from each group 2 weeks post treatment. Three contiguous slices of the tumor are shown. All treatment groups showed significant reduction in rBV compared to controls. Combination treatment resulted in the greatest reduction in tumor perfusion. *p<0.05, **p<0.01, ***p<0.001.

Next, we examined the effects of combined Dll4 and VEGF blockade on tumor vascular function using contrast-enhanced MRI. Tumors were imaged at 24 hours (early) and 2 weeks (late) after the start of treatment to characterize the vascular response of tumors to acute and chronic administration of both agents. Quantitative estimates of contrast agent concentration (ΔR1) in tumors (n = 3 per group) and contralateral kidneys were obtained to compute changes in blood volume (Y-intercept) and permeability (slope). At 24 hours post treatment (Fig. 5A), a significant reduction in blood volume (*p*<0.001) in tumors treated with ziv-aflibercept alone (0.25±0.06) or REGN1035 alone (0.72±0.10) compared to control tumors (1.08±0.09) was observed. Animals treated with the combination showed the greatest reduction in vascular volume compared to controls and either monotherapy (*p*<0.0001). Combination treatment resulted in durable anti-vascular activity with a significant (*p*<0.0001) reduction in blood volume (0.015±0.06) at the two week time point (Fig. 5B) compared to controls (0.75±0.05), ziv-aflibercept alone (0.14±0.06) and REGN1035 alone (0.20±0.04). No difference in perfusion (ΔR1) of kidneys was observed with single agent or combination treatments at both time points (Fig. S2). The panel of images shown in Figure 5C represent contrast enhancement maps of three contiguous slices of a tumor in each group at the end of two week treatment period. While single agent treatments resulted in reduction in perfusion compared to control tumors, combination treatment resulted in marked reduction in tumor growth and perfusion as evidenced by decreased contrast agent uptake in the tumor. Corresponding 3D MR angiography images of a representative tumor in all 4 groups is shown in Figure S3.

### Dll4 blockade enhances the anti-vascular effects of VEGF blockade in a sunitinib resistant RCC model

**Figure 6:**
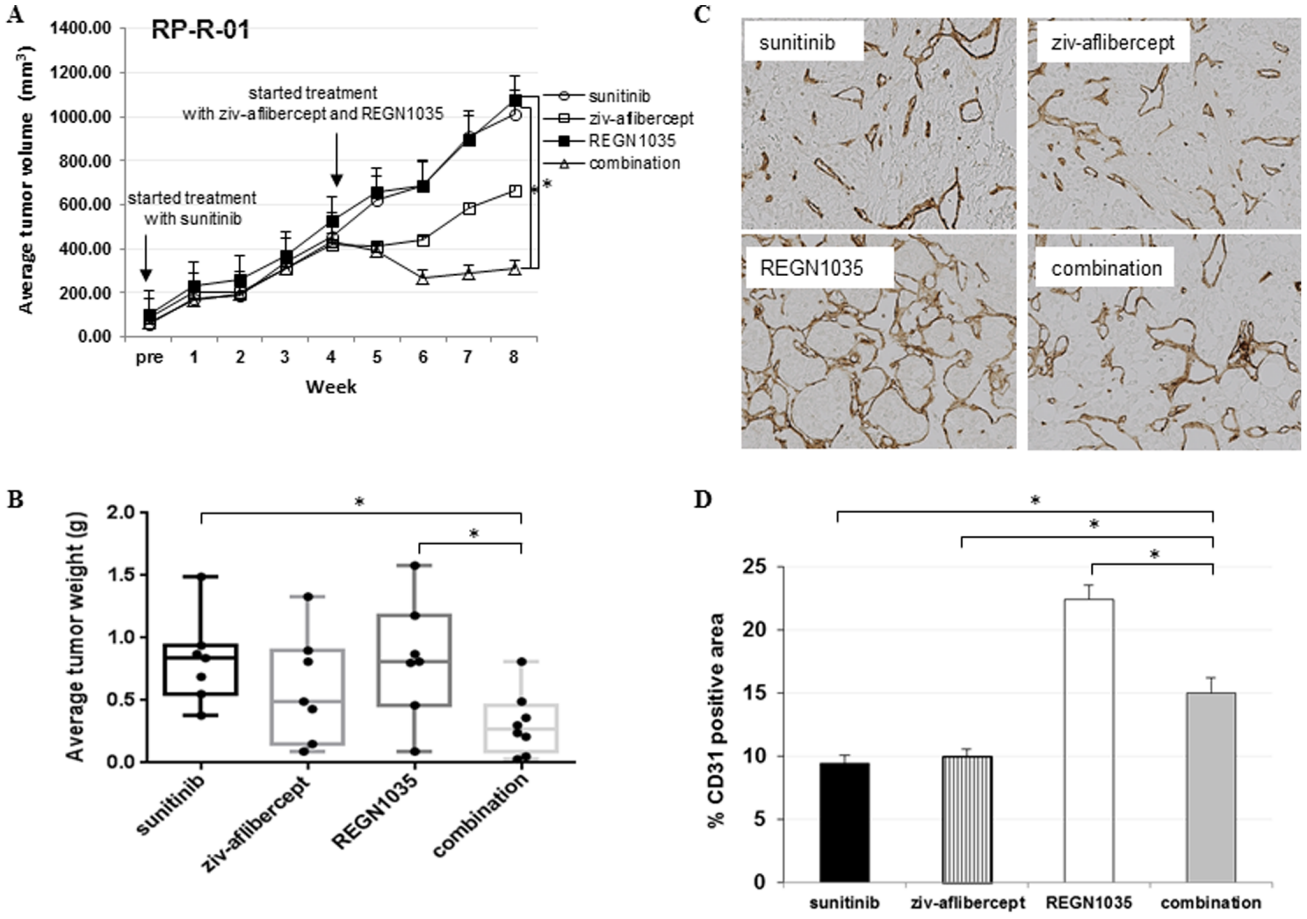
Anti-tumor efficacy of anti-Dll4 (REGN1035) and anti-VEGF (ziv-aflibercept) in a sunitinib resistant RP-R-01 ccRCC patient-derived xenograft model. Mice with established subcutaneous tumors were treated with sunitinib for 4 weeks until tumor tissue was no longer responsive to treatment (tumor volume was ∼6 times that of pretreatment volume). At which time, mice were treated with either sunitinib, ziv-aflibercept, REGN1035 or the combination of ziv-aflibercept plus REGN1035 for a period of four weeks. (A) Tumor growth curve of average tumor volume (mm^3^) ± S.E. (B) End point tumor weights (g). *p<0.05, as compared to combination group using adjusted t-test analysis. *Effect of REGN1035 and/or anti-VEGF (ziv-aflibercept) on sunitinib resistant RP-R-01 tumor vasculature*. Sunitinib resistant tumors from treated mice were harvested, processed, and tissue sections were stained for the differential expression of CD31. (C) Representative pictures. (D) Blind quantitative analysis of CD31. Results are expressed as mean percentage positive stained area ± S.E. *p<0.05, as compared to combination group using t-test analysis.

To assess the anti-tumor efficacy of anti-Dll4 and anti-Dll4/VEGF combination therapy in a sunitinib resistant xenograft model, SCID mice were implanted subcutaneously with RP-R-01 tissue and treated with sunitinib until resistance was observed (when tumor size doubled that of pretreatment size). At which time, mice either continued to be treated with sunitinib or were switched to ziv-aflibercept, REGN1035 or the combination of ziv-aflibercept plus REGN1035. As shown in Figure 6A, only the combination of REGN1035 and ziv-aflibercept induced a significant tumor growth inhibition and even tumor regression, as compared to the group continued to be exposed to sunitinib. EOT tumor volume and weight are shown in Figures 6A and 6B, respectively: sunitinib (1010.5 mm^3^, 0.823 g), ziv-aflibercept (661.8 mm^3^, 0.600 g), REGN1035 (1075.3 mm^3^, 0.827 g), and combination (308.9 mm^3^, 0.311 g). The anti-tumor efficacy of the combination treated mice was found to be significantly different (*p*<0.05) from single agent sunitinib and REGN1035 treated mice, but not ziv-aflibercept treated mice. Interestingly, the greater anti-tumor effect in the combination group was not associated with greater inhibition of tumor blood vessel density (Fig. 6C–D). No overt signs of toxicity were observed, with only minimal weight loss was noted in the combination group (6.35%, ±1.28).

### The potent anti-tumor activity of Dll4 blockade is dependent on targeting Dll4 in the tumor stroma

**Figure 7:**
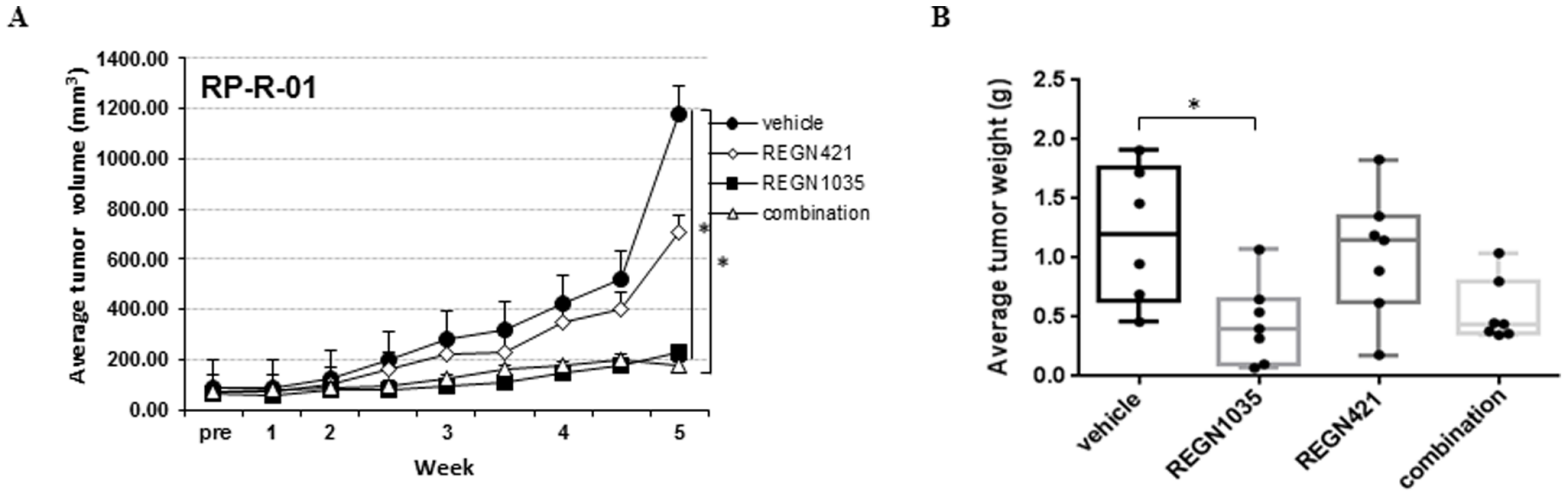
Effect of combined Dll4 blockade on RP-R-01 tumor and stromal cells. Mice were treated with vehicle, human Dll4-specific antibody REGN421 (10 mg/kg, 5x/wk, s.q.), mouse Dll4-specific antibody REGN1035 (5 mg/kg, 1x/wk, s.q.) or combination. (A) Tumor growth curve of treatment groups. Each line represents the average tumor volume (mm^3^) of each treatment group ± S.E. (B) Average end point tumor weights. *p<0.05, as compared to vehicle group; combination compared to single agents (REGN421, *p* = 0.160; REGN1035, *p* = 0.691) using adjusted t-test analysis.

Species-specific Taqman RNA gene expression analysis was performed to determine the levels of Dll4 expression in the RP-R-01 model and to gain a better understanding of the anti-tumor mechanism of anti-Dll4 therapy. Mouse Dll4 was robustly expressed in the stroma of RP-R-01 tumors, consistent with the function of Dll4 as a critical regulator of tumor angiogenesis, whereas the levels of tumor-cell expressed Dll4 (human) were very low, at the limit of detection (Fig. S4). To determine the relative contributions of blocking stromal (mouse) Dll4 vs. Dll4 expressed by tumor cells (human) to the overall anti-tumor activity, we treated RP-R-01 tumor-bearing SCID mice with the human Dll4-specific monoclonal antibody REGN421 (enoticumab), the mouse Dll4-specific antibody REGN1035, or the combination. As shown in Figures 7A and 7B, treatment with human Dll4-specific REGN421 showed only marginal anti-tumor efficacy, with no significant differences observed from the vehicle treated group. The mouse Dll4-specific REGN1035 treatment, on the other hand, resulted in significant RP-R-01 tumor growth inhibition (67%, *p*<0.01), consistent with results shown in Figure 1. Combination treatment of anti-human and anti-mouse Dll4 antibodies did not result in enhanced anti-tumor effects compared to single agent administration of REGN1035. These results demonstrate that the anti-tumor activity of Dll4 blockade in the RP-R-01 RCC model is entirely dependent on targeting Dll4 in the tumor stroma as opposed to tumor cell-expressed Dll4, and furthermore highlights the lack of tumor growth-promoting autocrine Dll4-Notch tumor cell signaling in this model.

## Discussion

The treatment options for patients with renal cell carcinoma have expanded considerably over the past decade, with five agents targeting VEGF signaling now approved for advanced disease. However, overcoming acquired resistance to frontline anti-VEGF therapies remains a challenge. Ample data in the literature indicate that Dll4 signaling plays a critical role in tumor angiogenesis and possibly in mediating resistance to anti-VEGF therapies, thus providing rationale for combined VEGF and Dll4 inhibition. Blockade of the Dll4-Notch pathway in preclinical cancer models results in non-productive angiogenesis, that is, excessive production of aberrant non-functional tumor vascular structures associated with reduced tumor growth. Thus, it functions via a different mechanism of action than the targeting of the VEGF signaling pathway. In fact, some tumors resistant to anti-VEGF therapy have been shown to be responsive to anti-Dll4 [10], [17]; and the inhibition of the Dll4-Notch signaling pathway has been found to enhance the efficacy of VEGF inhibitors [18]. Furthermore, patients with low Dll4 expression treated with anti-VEGF therapy were found to exhibit significant prolongation of progression free survival over patients with higher levels of Dll4 expression [19]. Similarly, high expression of Dll4 by endothelial cells was shown to be a statistically significant adverse prognostic factor in breast and ovarian cancer patients [20], [21]; and elevated expression of both DLL4 and VEGF was found to be a significant overall survival disadvantage in nasopharyngeal carcinoma patients, as compared to those with dual low expression [22]. Taken together, these findings provide a strong rationale for the combined blockade of VEGF and Dll4-Notch signaling in renal cancer patients.

Herein, we analyzed the anti-tumor activity of blocking Dll4-Notch signaling alone and in combination with anti-VEGF agents in patient-derived ccRCC models. We found that treatment with the anti-mouse Dll4 monoclonal antibody REGN1035 produces potent single agent anti-tumor efficacy in these models. Importantly, combination treatment with REGN1035 and either VEGF pathway targeting agents results in enhanced anti-tumor effects and even produced tumor regression.

Our study highlights the angiogenic mechanism by which blockade of Dll4 results in inhibition of tumor growth and vessel functionality. Extensive tumor necrosis and non-functional angiogenesis were observed in RCC tumors in response to Dll4 blockade. More specifically, a decrease in tumor perfusion was observed following REGN1035 treatment by MRI imaging in spite of the marked increase in tumor vessel density observed in these samples, confirming that Dll4 inhibition produces non-productive tumor angiogenesis that is associated with the subsequent impairment in tumor growth. Combination treatment of anti-Dll4 antibody with either anti-VEGF agent resulted in reductions in tumor perfusion and markedly increased tumor necrosis compared to single agents. Of note, analysis of the tumor vasculature revealed that the combination treatments produced decreases in microvascular densities similar to or exceeding the anti-vascular effects observed for the VEGF pathway targeting agents alone, suggesting that in the models analyzed in this study, the anti-VEGF vascular ‘pruning’ effects were dominant over the endothelial hypersprouting phenotype associated with Dll4 blockade.

In a separate experiment, we tested whether Dll4 blockade in combination with continuous VEGF inhibition could also impair tumor growth in a sunitinib resistant PDX model. Interestingly, despite clear blood vessel remodeling, Dll4 blockade did not have activity as a single agent in this sunitinib resistant PDX model, but it potentiated the anti-tumor effect of ziv-aflibercept by inducing tumor regression. However, the enhanced anti-tumor effect in the combination group was not associated with significant inhibition of blood vessel density as observed in the RP-R-01 sunitinib sensitive PDX model, suggesting, perhaps, that those tumor blood vessels, even if present, may be less functional. This additional observation supports the hypothesis that Dll4 blockade in the setting of effective VEGF inhibition may have clinical benefit also in tyrosine kinase inhibitor resistant ccRCC.

The Dll4-Notch signaling pathway has been implicated in regulating tumor initiating cells, also referred as cancer stem cells (CSCs) [23], [24]. This population of cells, which is reported to possess self renewal, tumor initiating, and differentiating properties, has been shown to play a role in tumor growth, metastasis, and tumor resistance [25]. In our experimental setting, we did not observe single agent anti-tumor activity for the selective targeting of human, tumor cell-expressed Dll4 or anti-tumor additivity for the concomitant blocking of human (tumor cells) and murine (endothelial cells) Dll4. Although, this finding does not rule out the possibility that targeting tumor (stem) cell-expressed Dll4 may contribute to the overall anti-tumor activity of Dll4 blockade in other settings, it suggests that the anti-tumor activity of Dll4 blockade in the employed RCC PDX model is dependent on targeting Dll4 in the tumor stroma, and the lack of tumor growth-promoting autocrine Dll4-Notch tumor cell signaling in this model.

Reports in the literature have raised some concern regarding the safety of chronic anti-Dll4 treatment [26], [27], but the majority of studies have shown it to be well tolerated. Dll4 expression is found to be extensive in the immature developing endothelium of neoplastic tissue, with low to undetectable levels in normal tissue, which constitutes the basis for the specific targeting of the tumor endothelium. While we observed some weight loss (<10%) in mice treated with REGN1035, sunitinib or REGN421 on an acute treatment schedule (See Table S2), the animals did not present overt signs of toxicity. We did observe a 16% body weight loss in the REGN1035-ziv-aflibercept combination group. However, it should be noted the vehicle group also had a 10% decrease in body weight and the difference with the combination group was not statistically significant (Table S2). Histological examination of treated livers revealed mild evidence of septal and periportal fibrosis and vascular congestion (Fig. S1). In addition, the observed increase in liver blood vessels induced by single agent REGN1035 was modest as compared to the effect on tumor blood vessels. Overall, our data suggest that this combinatorial treatment approach is relatively well tolerated in the preclinical models utilized, though we recognize that the time of drug exposure was limited to clearly define the toxicity of this combination. This is important in view of the fact that combinations of VEGF inhibitors with other targeted therapies (i.e. mTOR inhibitors) in RCC have been hampered by increased toxicity and lack of clear greater clinical benefit as compared to single agents. Dll4 blockade may represent an alternative target for therapeutic interventions in combination with either a VEGF blocker (i.e. bevacizumab or ziv-aflibercept) or a VEGF receptor tyrosine kinase inhibitor (i.e. sunitinib, pazopanib or axitinib) to delay the occurrence of acquired-resistance to approved VEGF inhibitors.

In conclusion, we report here the potent anti-tumor activity for the blockade of Dll4 as monotherapy and a benefit for the combined treatment of anti-Dll4 with VEGF pathway targeting agents in ccRCC PDX models. Our data provide support for combining anti-angiogenic therapies to achieve a more effective treatment response; and specifically warrant clinical investigation of anti-Dll4 therapies alone and in combination with VEGF targeting agents for the treatment of ccRCC.

## Supporting Information

Figure S1 **Liver H&E histology.** (A) Mice inoculated with RP-R-02 tumor tissue were treated for 5 weeks with vehicle, REGN1035, or REGN1035 plus ziv-aflibercept combination. Livers were harvested, processed, and tissue sections were stained for hematoxylin and eosin. Representative image of (left) vehicle treated mice shows normal histology, (middle) REGN1035 treated shows mild septal fibrosis and vascular congestion, and (right) REGN1035 plus ziv-aflibercept also shows mild congestion, periportal fibrosis, and micro steatosis. *Effect of anti-Dll4 and/or anti-VEGF (ziv-aflibercept) on liver vasculature*. (B and C) RP-R-R01 bearing mice previously exposed to sunitinib were treated for 4 additional weeks with sunitinib, REGN1035, or REGN1035 plus ziv-aflibercept combination (See experiment in Fig. 6). Livers from treated mice were harvested, processed, and tissue sections were stained for the differential expression of CD31. Quantitative analysis of CD31 staining was performed in a blinded fashion. Results are expressed as mean percentage positive stained area ± S.E. *p<0.05, as compared to single agent anti-Dll4 (REGN1035) group using t-test analysis.
(TIF)

Figure S2 **Tumor and kidney perfusion.** (A and B) Plots show the change in R1 (ΔR1) values of tumors 24 hours and 2 weeks post therapy, respectively. (C and D) No difference in perfusion (ΔR1) of kidneys (normal) was observed with single agent or combination treatments at both time points highlighting the selectivity of tumor vascular response to therapy.
(TIF)

Figure S3 **Contrast-enhanced 3D MR angiography images of mice bearing RP-R-01 tumors (outlined in yellow) from all four treatment groups (vehicle, ziv-aflibercept, REGN1035, and combination) at both time points.** Control tumors showed marked signal enhancement following contrast administration at both time points (24 hours and two weeks), indicative of the well vascularized nature of these tumors. Control tumors also showed increased growth over the two week period. While single agent treatment with ziv-aflibercept and REGN1035 resulted in moderate tumor growth inhibition and reduction in perfusion, combination treatment resulted in a significant reduction in tumor volume and perfusion as evidenced by a lack of contrast enhancement on the 3D angiography images.
(TIF)

Figure S4 **Dll4 expression in RP-R-01 tumors.** Dll4 expression in RP-R-01 tumors normalized to cyclophilin by TaqMan PCR. Mouse Dll4 is robustly expressed in the stroma of RP-R-01 tumors, while human Dll4 levels are at the limit of detection (mouse Dll4 mRNA is 780 times more abundant than human).
(TIF)

Table S1 **Statistical analysis of the tumor growth data.**
(XPS)

Table S2 **Average percent body weight change.** Mice were weighed weekly and the average percent change from baseline was calculated as follows: (End body weight - start body weight)/start body weight x 100± S.E.
(XPS)
