# Supplementary figures and images for: Dll4 Blockade Potentiates the Anti-Tumor Effects of VEGF Inhibition in Renal Cell Carcinoma Patient-Derived Xenografts
Source: PLoS One. 2014 Nov 13;9(11):e112371. doi: 10.1371/journal.pone.0112371 (PMC4231048; doi:10.1371/journal.pone.0112371)

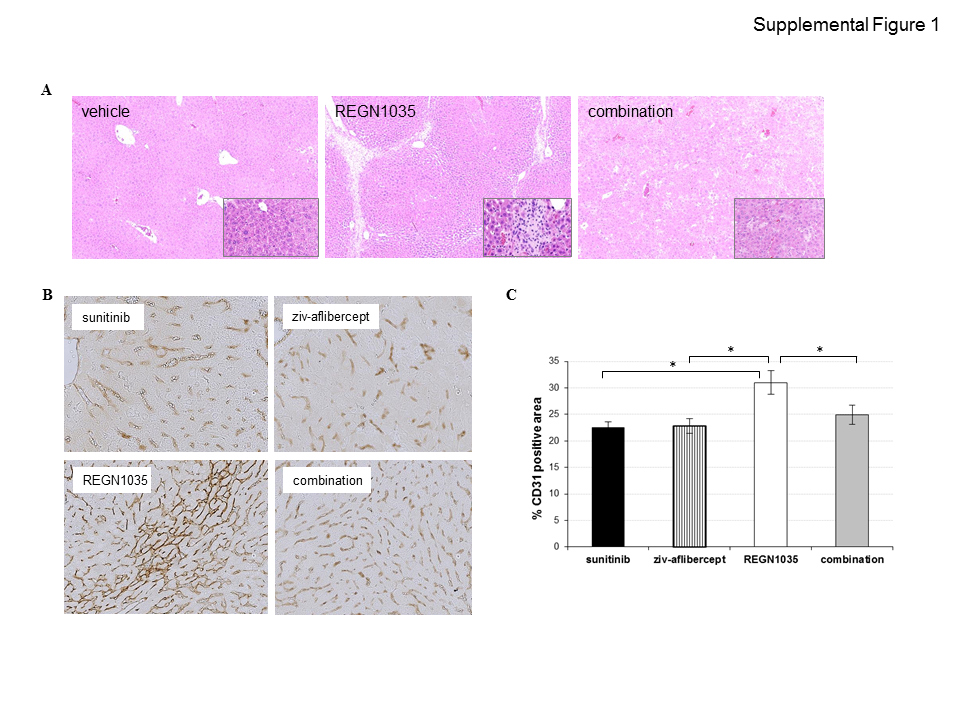

Supplement: Figure S1 — Liver H&E histology. (A) Mice inoculated with RP-R-02 tumor tissue were treated for 5 weeks with vehicle, REGN1035, or REGN1035 plus ziv-aflibercept combination. Livers were harvested, processed, and tissue sections were stained for hematoxylin and eosin. Representative image of (left) vehicle treated mice shows normal histology, (middle) REGN1035 treated shows mild septal fibrosis and vascular congestion, and (right) REGN1035 plus ziv-aflibercept also shows mild congestion, periportal fibrosis, and micro steatosis. Effect of anti-Dll4 and/or anti-VEGF (ziv-aflibercept) on liver vasculature. (B and C) RP-R-R01 bearing mice previously exposed to sunitinib were treated for 4 additional weeks with sunitinib, REGN1035, or REGN1035 plus ziv-aflibercept combination (See experiment in Fig. 6). Livers from treated mice were harvested, processed, and tissue sections were stained for the differential expression of CD31. Quantitative analysis of CD31 staining was performed in a blinded fashion. Results are expressed as mean percentage positive stained area ± S.E. *p<0.05, as compared to single agent anti-Dll4 (REGN1035) group using t-test analysis. (TIF) [file pone.0112371.s001.tif]

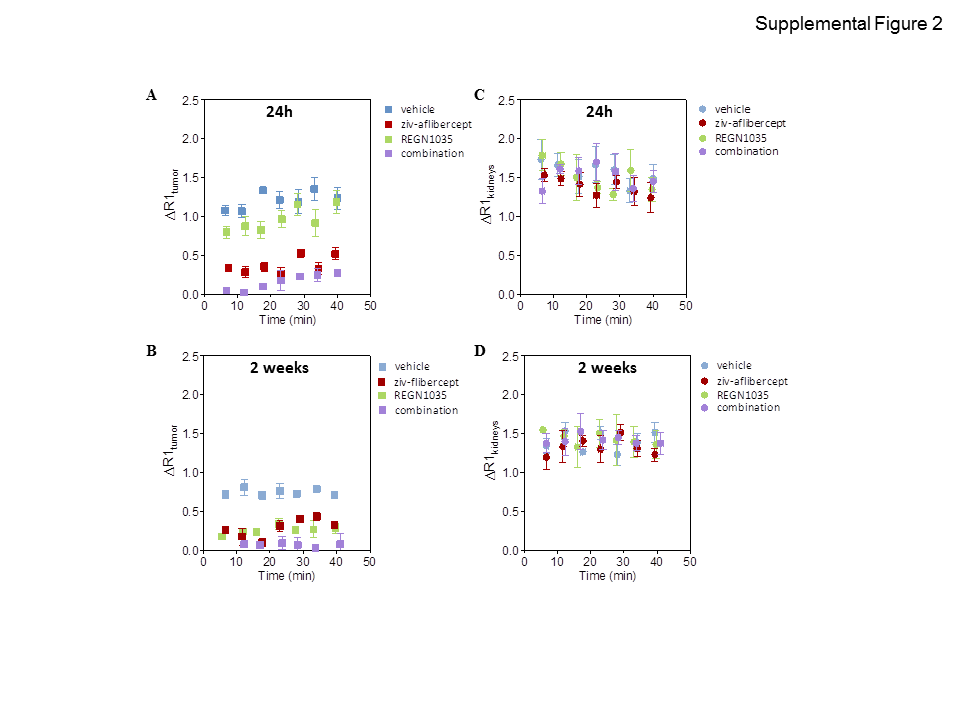

Supplement: Figure S2 — Tumor and kidney perfusion. (A and B) Plots show the change in R1 (ΔR1) values of tumors 24 hours and 2 weeks post therapy, respectively. (C and D) No difference in perfusion (ΔR1) of kidneys (normal) was observed with single agent or combination treatments at both time points highlighting the selectivity of tumor vascular response to therapy. (TIF) [file pone.0112371.s002.tif]

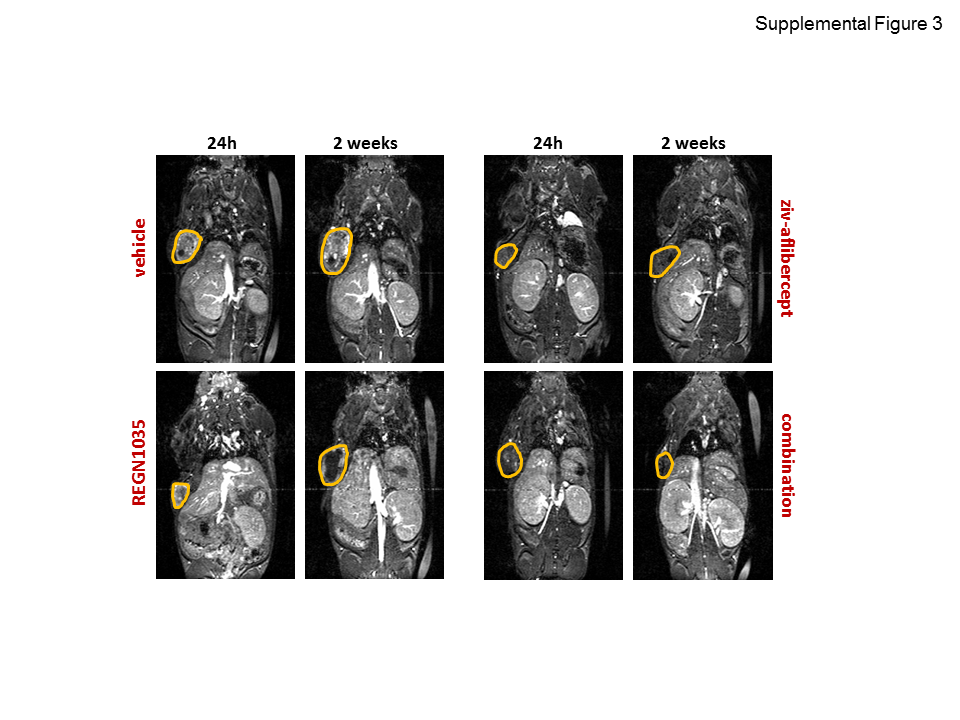

Supplement: Figure S3 — Contrast-enhanced 3D MR angiography images of mice bearing RP-R-01 tumors (outlined in yellow) from all four treatment groups (vehicle, ziv-aflibercept, REGN1035, and combination) at both time points. Control tumors showed marked signal enhancement following contrast administration at both time points (24 hours and two weeks), indicative of the well vascularized nature of these tumors. Control tumors also showed increased growth over the two week period. While single agent treatment with ziv-aflibercept and REGN1035 resulted in moderate tumor growth inhibition and reduction in perfusion, combination treatment resulted in a significant reduction in tumor volume and perfusion as evidenced by a lack of contrast enhancement on the 3D angiography images. (TIF) [file pone.0112371.s003.tif]

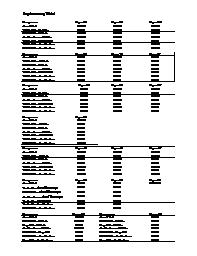

Supplement: Table S1 — Statistical analysis of the tumor growth data. (XPS) [file pone.0112371.s005.xps › docProps/thumbnail.jpeg]

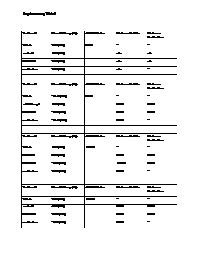

Supplement: Table S2 — Average percent body weight change. Mice were weighed weekly and the average percent change from baseline was calculated as follows: (End body weight - start body weight)/start body weight x 100± S.E. (XPS) [file pone.0112371.s006.xps › docProps/thumbnail.jpeg]
